# Bacterial Protein Toxins as Anticancer Agents: Clinical Potential of *Pseudomonas* and Anthrax Toxins

**DOI:** 10.3390/toxins17090459

**Published:** 2025-09-12

**Authors:** Richa Misra, Radhika Gupta, Namita Nayyar, Ritvik Baweja, Vishal Sharma, Yogendra Singh, Renu Baweja

**Affiliations:** 1 Department of Zoology, Sri Venkateswara College, University of Delhi, Delhi 110021, India; richamisra@svc.ac.in (R.M.); namitanayyar@svc.ac.in (N.N.); 2 Department of Biochemistry, Daulat Ram College, University of Delhi, Delhi 110007, India; radhikagupta@dr.du.ac.in; 3 Maulana Azad Medical College, Delhi 110002, India; ritvikbaweja@gmail.com; 4 Department of Infectious disease, Albert Einstein College of Medicine, New York, NY 10461, USA; s.vishalzoology@gmail.com; 5 Delhi School of Public Health, Institution of Eminence, University of Delhi, Delhi 110007, India; 6 Department of Biochemistry, Shivaji College, University of Delhi, Delhi 110027, India

**Keywords:** anthrax toxin, PE toxin, protein toxin, AB toxin, cancer therapy, immunotoxin

## Abstract

Protein toxins are biologically active polypeptides produced by a variety of organisms, including bacteria, plants, fungi, and animals. These molecules exert potent and specific toxic effects on target cells and are primarily associated with pathogenicity and defense mechanisms of the organisms. In the past few decades, significant progress has been made in understanding their structure, mechanisms of action, and regulation. Among these, bacterial protein toxins have emerged as valuable tools particularly in the development of targeted therapies. A notable example is Botulinum toxin, originally known for its neurotoxic effects, which was approved as a therapeutic agent in 1989 for strabismus treatment, paving way for repurposing bacterial toxins for clinical use. This review provides an overview of the different classes of bacterial toxin-based therapeutics, with a particular focus on *Pseudomonas* exotoxin A (PE) from *Pseudomonas aeruginosa* and anthrax toxin from *Bacillus anthracis*. The modular architecture and potent cytotoxicity of these A-B type toxins have enabled their successful adaptation into targeted cancer therapies. The clinical approval of the PE-based immunotoxin, moxetumomab pasudotox, for the treatment of hairy cell leukemia, underscores the potential of this strategy. This review also discusses current challenges and outlines future directions for the advancement of bacterial toxin-based therapeutics.

## 1. Introduction

Bacterial toxins belong to a large, biochemically diverse family of biotoxins produced by numerous living organisms, including plants (phytotoxins), fungi (mycotoxins), and animals (zootoxins). Bacterial toxins, traditionally recognized for their pathogenic roles in several infectious diseases, have increasingly been investigated as potential therapeutic agents owing to their ability to target specific cells, regulate immune responses, or induce cell death [1]. These toxins are broadly classified into two categories, namely, exotoxins and endotoxins. Exotoxins are peptide-based toxins which often exert lethal effects at very low concentrations [2]. Their properties such as heat resistance, poor stability, and strong antigenicity determine their specific mode of action and are linked to the development of various diseases [2,3]. These protein toxins are capable of manipulating many cellular processes and signaling pathways such as vesicle trafficking, protein synthesis, and cytoskeletal organization. In contrast, endotoxins, the lipopolysaccharide (LPS) molecules present on the outer membrane of Gram-negative bacteria are released only upon bacterial cell lysis. Once released into host tissues, they activate innate immune response, which is not preferred therapeutically due to its non-specificity [3].

**Figure 1:**
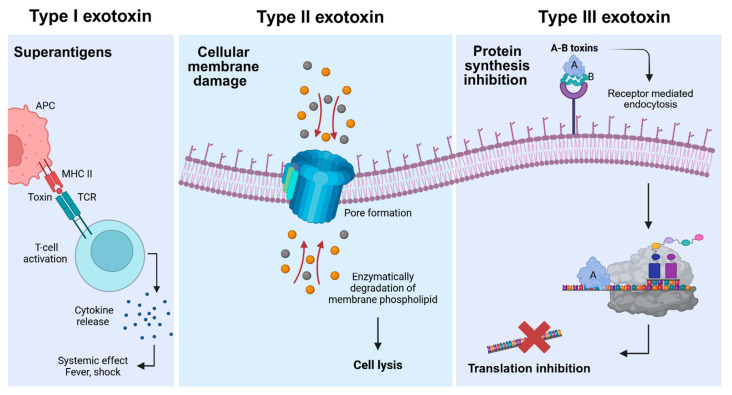
Classification of bacterial exotoxins into types I, II, and III. Type I exotoxins (**left panel**) bind simultaneously to major histocompatibility complex (MHC) class II molecules on antigen-presenting cells (APC) and T-cell receptors (TCR), leading to non-specific T-cell activation and excessive cytokine release. This cascade can result in systemic inflammatory responses, including fever and shock. Type II exotoxins (**middle panel**) disrupt cellular membranes by either forming proteinaceous pores or enzymatically degrading membrane phospholipids, causing the loss of cell integrity and ultimately cell lysis. The circles indicate uncontrolled ion flow though the pore, disrupting cellular homeostasis leading to cell death. Type III exotoxins (**right panel**) consist of a catalytic domain (A domain) and receptor-binding domain (B domain). Once inside the cell through the receptor-mediated endocytosis, a subset of A domains inhibits protein synthesis leading to cell death.

Exotoxins exert their effects through the distinct and potent mechanisms of action on host cells. They are classified into three categories on the basis of their structure and functions (Figure 1). Type I exotoxins, or superantigens, bind non-specifically to MHC (Major Histocompatibility Complex) class II molecules and T-cell receptors on the surface of antigen-presenting cells (APCs), non-specifically activating large number of T-cells and leading to a cytokine storm. Elevated levels of cytokines such as IL-2 in the blood can trigger a range of symptoms such as fever, shock, or even death. Examples include toxic shock syndrome due to the toxin produced by *Staphylococcus aureus* [1]. Type II exotoxins, or membrane-damaging toxins, target cell membranes directly, either by forming pores (e.g., alpha-hemolysin from *S. aureus*) or by enzymatically degrading membrane phospholipids (*Clostridium perfringens* α-toxin), leading to cell lysis [4,5]. Type III exotoxins, known as A-B toxins, consist of two components, where the B subunit (binding/translocation subunit) binds to specific host receptors and enables the entry of the enzymatic A subunit (active subunit). This active component then modifies intracellular targets to disrupt essential processes like protein synthesis and induces cell death. Classic examples include diphtheria toxin, cholera toxin, *Pseudomonas* exotoxin, and anthrax toxin [1,6].

These exotoxins by varied mechanisms disrupt host cell integrity and function, contributing significantly to bacterial pathogenicity. Of these exotoxins, AB toxins have garnered considerable interest as promising tools in therapeutic applications, specifically cancer therapy. Their structural diversity and unique mechanisms of action allow them to modulate host-specific targets, disrupt signaling pathways, and induce cell death [7]. Further classifications or sub-types can be obtained based on structural organization (single-chain versus multimeric/binary), cellular target (neurotoxins, cytotoxins, hemotoxins), and type of enzymatic activity (ADP-ribosylation, protease, adenylate cyclase, etc.), each conferring different pathophysiological or therapeutic properties. A more comprehensive understanding of these toxins is crucial for devising effective strategies, including vaccines and therapies, to combat bacterial infections and harness their potential for treating human diseases [1,7,8].

The therapeutic repurposing of bacterial toxins traces back to early 20th century, when William Coley administered cancer patients with a formulation of heat-killed *Streptococcus pyogenes* and *Serratia marcescens* bacteria (Coley’s toxins). This marked the first clinical attempts to treat cancer with bacterial products, laying the groundwork for immunotherapy [9]. Subsequent breakthroughs in structural biology and recombinant DNA technology enabled researchers to dissect toxin domains, engineer safer versions, and fuse them with targeting molecules for therapeutic purposes. The first bacterial toxin which received clinical approval for treating eye muscle disorder strabismus in 1989 was botulinum toxin from *Clostridium botulinum* [10]. The development of denileukin diftitox (Ontak), a diphtheria toxin-based immunotoxin, for cutaneous T-cell lymphoma in 1999 became the first successful example of developing an anti-cancer agent based on toxin moiety [11]. Immunotoxins are chimeric proteins created by coupling a receptor-binding moiety, typically an antibody or ligand targeting a specific cell-surface receptor, and catalytic moiety of a toxin, an enzyme, responsible for the toxin-induced lethality [12]. Cancer is a leading cause of mortality throughout the world and despite significant medical advancements in the treatment of cancer, targeting and treating certain cancer types is extremely challenging [13]. Immunotoxins have been particularly promising in treating cancer cells by targeting specific types of cells.

**Figure 2:**
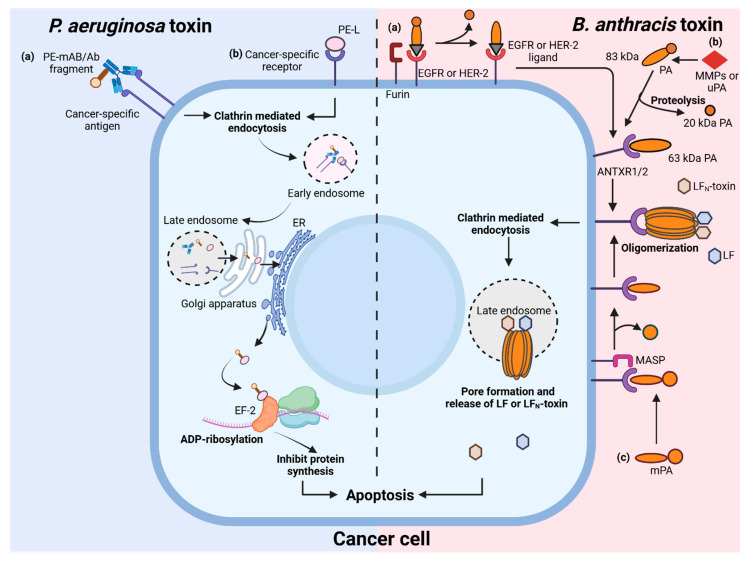
Schematic overview of *Pseudomonas aeruginosa* and *Bacillus anthracis* toxins in targeting cancer cells. *P. aeruginosa* toxin pathway (**left panel**): cancer-specific antibody fragments (a) or ligand (b) fused with *Pseudomonas* exotoxin (PE) target tumor-associated antigens or receptors. The toxin enters the cell through clathrin-mediated endocytosis and is transported via endosomes to the endoplasmic reticulum (ER) through the Golgi apparatus. In the ER, the active portion of the exotoxin is released into the cytosol where it catalyzes the ADP-ribosylation of elongation factor 2 (EF-2), resulting in the inhibition of protein synthesis and induction of apoptosis. *B. anthracis* toxin pathway (**right panel**) illustrates various strategies for cancer-specific targeting by anthrax toxin variants. (a) Protective antigen (PA) can be fused or complexed with ligands or antibody fragments that bind to receptors commonly overexpressed on tumor cells (such as EGFR or HER2). Upon binding, cell-surface proteases like furin cleave PA, initiating toxin activation; (b) PA activation no longer depends on ubiquitous furin, but on tumor-associated proteases such as matrix metalloproteinases (MMPs) or urokinase-type plasminogen activator (uPA), which are hyperactive in the tumor microenvironment. Alternatively, (c) modified PA (mPA) is recognized and cleaved by membrane anchored serine proteases (MASPs), overexpressed on cancer cells. Cleaved protective antigen (PA) associates with anthrax toxin receptors (ANTXR1/2) and oligomerizes, then recruits lethal factor (LF). The complex undergoes clathrin-mediated endocytosis, enters late endosomes, and then forms a pore through which LF is released into the cytosol. LF disrupts critical signaling, resulting in cancer cell apoptosis. Created with BioRender.com.

One of the most widely studied toxins for cancer therapy includes the exotoxin encoded by *Pseudomonas aeruginosa*, known as *Pseudomonas* exotoxin A (PE), which functions through a well-defined mechanism. Briefly, the toxin enters the target cell through receptor-mediated endocytosis, followed by its activation via furin cleavage. This active toxin then enters the cytosol, where it inhibits protein synthesis through the ADP-ribosylation of the eukaryotic elongation factor-2 (eEF-2), ultimately inducing apoptosis (Figure 2). The toxin is divided into multiple domains, each with specific roles in binding, translocation, and enzymatic activity [14,15]. Its high potency, along with ease of expression and purification, has made PE a valuable candidate in the development of cancer-targeting immunotoxins, where it is fused or conjugated with monoclonal antibodies (mAbs) or ligands specific to cancer cells [15,16].

Another promising example of a bacterial toxin now being repurposed as a precision tool in therapeutics is the toxin produced by *Bacillus anthracis* and is called anthrax toxin. Briefly, the bacterium gains entry into the host macrophages through its spores, which germinate and produce toxin. The toxin consists of three components, protective antigen (PA), edema factor (EF), and lethal factor (LF). PA binds to host cell receptors and is cleaved by furin proteases to form a heptameric/octameric oligomer. This oligomer binds LF and/or EF and is internalized into host cells via clathrin-mediated endocytosis. Acidification of the endosome triggers PA pore formation, allowing the translocation of LF and EF into the cytoplasm, which mediates anthrax pathogenicity (Figure 2). The evolution of the anthrax toxin mechanism, originally contributing to bacterial pathogenesis, has become the basis for transforming anthrax toxin into a precise anti-cancer delivery system [17,18,19]. The following sections will discuss the structures, mechanisms, engineered variants, and clinical journey of *Pseudomonas* and anthrax toxins in this dynamic anti-cancer therapeutic landscape.

## 2. *Pseudomonas aeruginosa* Toxin-Based Therapeutics

*P. aeruginosa* is a Gram-negative bacterium and causes infection in immuno-compromised individuals. *P. aeruginosa* produces many toxins which include exotoxins (ExoA or PE, ExoT, ExoS, ExoU), endotoxin (LPS released on cell lysis), and many toxic lipids and enzymes [20]. Among these, PE has become the most widely used in therapeutics, owing to its potent, specific, and modular activity, making it ideal for therapeutic engineering, especially in the form of immunotoxins for targeted cancer treatment. In contrast, ExoS, ExoT, and ExoU are important virulence factors but are harder to repurpose for safe therapeutic use due to less adaptability. PE is a single chain peptide AB toxin (66 kDa) consisting of 638 amino acids (aa) organized into three domains: domain I is the receptor binding domain and it consists of domain Ia (1–252 aa) and domain Ib (365–404 aa), respectively; domain II (253–364 aa) is required for translocating the catalytic domain in the cytoplasm; and third domain is the C-terminal catalytic domain and contains the endoplasmic reticulum retention sequence that mediates retrograde transport of PE from the Golgi to the ER (domain III 405–613 aa) [15,21]. The catalytic domain has ADP-ribosylating activity which transfers ADP-ribose to elongation factor 2 (EF2). This inhibits protein synthesis, ultimately causing cell death. The molecular structure of PE wildtype is accessible via PDB code 1IKQ [14,22]. PE contains an N-terminal 25-residue hydrophobic signal sequence which is cleaved during secretion, resulting in a mature toxin of 613 aa [23]. The mature toxin binds to the alpha 2-macroglobulin receptor (CD91) on the host cell surface primarily via domain Ia and is internalized through receptor-mediated endocytosis [24]. Upon endosomal acidification, PE undergoes conformational changes that cause it to disassociate from the receptor and expose a furin cleavage motif located between arginine 279 and glycine 280 in domain II [22,25]. The host protease furin cleaves PE at this site, but the cleaved products remain connected by disulphide bonds between cysteine residues at 265 and 287 positions. This bond is further reduced by protein disulphide isomerases, generating a 37 kDa enzymatically active protein [26]. The C-terminus lysine residue at position 613 of PE is cleaved by carboxypeptidases, converting the REDLK motif (609–613 aa) to REDL (609–612 aa), which facilitates binding to KDEL receptors at the Golgi apparatus [27]. Through retrograde transport mediated by Rab proteins, the toxin enters the Golgi apparatus via the trans-Golgi network and then transported to the endoplasmic reticulum (ER) [28,29]. The active toxin fragment is then translocated from the ER into the cytoplasm through the ER-associated protein degradation (ERAD) pathway, utilizing the Sec61p translocon channel [30,31]. Once in the cytoplasm, the catalytic domain of PE ADP-ribosylates the eEF-2 through a SN1 nucleophilic substitution mechanism [32,33]. This process begins with the binding of PE to the NAD^+^ moiety of eEF-2 at the active loop (aa 483–490) of domain III of toxin [34]. The glycosidic bond between sugar and nicotinamide of NAD^+^ is cleaved, generating a reactive oxacarbenium ion intermediate stabilized by glutamate at position 553 of PE [33,35]. The post-translationally modified histidine residue (diphthamide, (2-(3-carboxyamido-3-[trimethylammonio]propyl) histidine) on eEF-2 nucleophilically attacks this intermediate, transferring the ADP-ribose group to the imidazole ring and resulting in the ADP-ribosylation of eEF-2 [33,36,37]. Since eEF-2 mediates the GTP-dependent translocation of mRNA from site A to site P of the ribosome, its inactivation results in complete stalling of protein synthesis, ultimately leading to cell apoptosis [38,39]. The resulting translational shutdown upregulates pro-apoptotic BH3 proteins, leading to mitochondrial dysfunction, and the activation of caspase cascades through cytochrome c release [40].

PE is a highly effective immunotoxin scaffold, favored for its high cytotoxicity, ease of genetic manipulation, low non-specific toxicity, and efficient expression and purification. The concept of cancer immunotherapy is based on Paul Ehrlich’s magic bullet theory, which was conceptualized by the discovery of mAbs, as these can be targeted to specific tumor cells. Several immunotoxins have been prepared by coupling PE to mAbs, growth factors, or cytokines. These immunotoxins are designed such that they can specifically target cancer cells with minimal effects on normal tissue. The first PE-based immunotoxin was developed in 1985, when full-length PE was chemically linked to ovarian cancer-specific mAbs (OVB3) [41]. Although these immunotoxins were effective in killing ovarian cancer cells in vitro and in clinical trials, they induced notable side effects due to non-specific binding and immunogenicity. Neutralizing antibodies (NAbs) developed against immunotoxin within 2 weeks of administration, limiting therapeutic efficacy. These findings underscored the need to develop efficacious therapeutic agents to improve specificity, reduce immunogenicity and cause minimal side effects. Hence, over the years, various engineered PE derivatives have been developed by the structural optimization and humanization of the PE immunotoxin improving therapeutic index and clinical utility.

Various types of PE proteins variants include the following:

- Full length PE: The earliest immunotoxins used intact PE, chemically coupled to OVB3 mAbs (specific to ovarian cancer). These showed dose-dependent neurotoxicity (by OVB3 antibodies) and hepatotoxicity (by binding PE via domain I to the hepatocytes) with the development of antibodies against the immunotoxin within 2 weeks of injection in ovarian cancer patients [41].
- LysPE40: After structural optimization, this truncated version of PE was generated. The domain Ia (aa 1–252) was removed with an added lysine residue at the amino terminus for effective antibody coupling. PE40 was 200-fold less toxic to mice as compared to full length PE [42].
- PE38: This version of PE was further truncated by removing both domain Ia (aa 1–252) and a portion of domain Ib (aa 365–380). The removal of non-essential aa 365–380 eliminated T-cell epitopes from PE40, thus reducing immunogenicity. To decrease the non-specific toxicity of PE, a recombinant protein PElys38 was generated. B3 mAbs against ovarian cancer were chemically coupled to lysPE38 to generate LMB-1, effective against colon and breast cancers. However, immune responses and vascular leak syndrome (VLS) limited its clinical application and further versions of the immunotoxin were developed [43].
- Recombinant chimeric PE: The next generation of PE was developed by fusing the truncated PE toxin gene with antibody gene on a single DNA construct, instead of chemical conjugation. The single-chain immunotoxin versions of OVB3-PE and LMB-1 (B3-LysPE38) were less immunogenic, cost effective, and easy to produce in *Escherichia coli*. They also had better tumor penetration due to their smaller size [44]. The LMB-1 trial was discontinued due to VLS and the formation of NAbs.
- KDEL-modified variants (PE40KDEL, PE38KDEL, and LysPE38KDEL): The native REDL sequence at the C-terminus was replaced by KDEL sequence in PE 40 and PE38, which enhanced ER retention and the cytotoxicity of toxin. PE40KDEL was fused to the Fv portion of the anti-Tac mAb targeting CD25, which is highly expressed on T-cell leukemia cells. However, in murine models, the administration of this immunotoxin resulted in VLS [45]. To overcome this, PE38KDEL was developed, a further truncated and less immunogenic variant, which was fused to single chain variable fragment (scFv) of the anti-Tac mAb to create LMB-2. This construct exhibited improved specificity, reduced toxicity, and enhanced anti-tumor activity. Similarly, LMB-7 was engineered by making a chimera of lysPE38KDEL (a version with an additional N-terminal lysine for improved conjugation) with scFv of B3 mAb. Although LMB-7 was effective in the immunodeficient mouse models of epidermoid carcinoma, its clinical progress was hindered by intracellular aggregation [46]. A more recent construct, D2C7-(scdsFv)-PE38KDEL (D2C7-immunotoxin), was developed by fusing a disulfide stabilized scFv of D2C7 with PE38KDEL. This design aimed to enhance structural stability and tumor-targeting precision, particularly for glioblastoma models expressing the wild-type epidermal growth factor receptor (EGFR) and EGFRvIII.
- PE38QQR: This is a PE38-optimized immunotoxin with increased toxicity. The PE38QQR toxin was generated by replacing lysine 590 and lysine 606 of PE with glutamines and lysine 613 with arginine. At the N-terminus, a lysine residue was added for antibody coupling. PE38QRR had a better synthetic yield, increased intracellular retention, enhanced cytotoxicity, and lower hepatotoxicity [47]. The immunotoxin was taken for a Phase III trial called PRECISE with 256 patients where intratumoral therapy was provided for high-grade gliomas. As expected, the immunotoxin was well tolerated when administered via convention-enhanced delivery but had no survival advantage [48].
- PE35: Domain II (aa 253–279) was removed from PE38KDEL to generate PE35, lacking disulfide linkages and T-cell epitopes, thus lowering immunogenicity. The PE35/TGFα-KDEL immunotoxin targeted EGFR-bearing bladder carcinomas with improved tolerance [49].
- PE24: A highly minimized form retaining only the furin cleavage site and catalytic domain III, eliminating domain I and domain II entirely. This variant helped in achieving maximum efficacy with minimal side effects such as hepatotoxicity and VLS. It forms the basis of LMB-110, which is generated by fusing PE24 with a fully humanized anti-mesothelin Fab. A single cycle of LMB-100 led to a marked reduction in lung carcinoma in a murine model [50]. In phase I trials, LMB-100 has shown promising results in mesothelioma and other solid tumors expressing mesothelin [50].
- PE25X6/PE25X7: These are next-generation immunotoxins with six or seven site-directed mutations in domain III to eliminate B-cell and T-cell epitopes while preserving activity. They retain only the furin site from domains I/II, significantly reducing immunogenicity and off-target effects [51].

These efforts demonstrate how the iterative engineering of PE has led to safer, more effective immunotoxins. These PE variants differ in their domain compositions and C-terminal sequences to enhance intracellular retention and minimize off-target effects. Numerous immunotoxins derived from these PE variants—such as LMB-1, LMB-2, LMB-7, SS1P, and the advanced LMB-100—have progressed through preclinical evaluation and clinical trials for a range of malignancies, including leukemia, ovarian cancer, pancreatic cancer, breast cancer, glioblastoma, and mesothelioma. Figure 2 schematically describes mechanism of the cellular uptake, intracellular trafficking, and cytotoxic effects of engineered PE used in cancer therapy.

A comprehensive list of recent PE-based clinical trials is provided in Table 1.

Despite their success at inducing tumor regression, PE immunotoxins have faced persistent obstacles: NAbs, VLS, and limited long-term efficacy due to the emergence of resistance mechanisms, leading to the ongoing refinement of their structure and delivery strategies [74]. Nonetheless, the clinical approval of PE-based moxetumomab pasudotox validates the potential of this approach and PE-based immunotoxins continue to evolve as promising agents in oncologic drug development.

## 3. *Bacillus anthracis* Toxin-Based Therapeutics

*B. anthracis*, a spore-forming, Gram-positive bacterium, is the causative agent of anthrax in humans and other animals. Anthrax is primarily a zoonotic disease, with humans serving as incidental hosts who become infected through direct or indirect contact with the diseased animals. The main infectious form is its highly resistant spores, which can enter the body via cutaneous, inhalational, gastrointestinal, or injection routes. Once inside the host macrophages, spores germinate to produce anthrax toxin, the major factor responsible for bacterium’s virulence.

Anthrax toxin is a three-component exotoxin: PA, EF, and LF. Individually, these toxin components are non-toxic, but when PA combines with EF or LF, it forms edema toxin (EdTx) and lethal toxin (LeTx), respectively, which are primarily responsible for pathogenicity of anthrax. Briefly, PA, an 83 kDa protein, binds to one of the two ubiquitously expressed receptors, namely anthrax toxin receptor 1 (ANTXR1) or TEM8 (tumor endothelial marker-8) and anthrax toxin receptor 2 (ANTXR2) or CMG2 (capillary morphogenesis gene-2), transmembrane receptors present on the host cell surface [75,76,77,78]. Following receptor binding, a 23 kDa N-terminal fragment of PA is cleaved by a calcium-dependent furin endoprotease, yielding a 63 kDa fragment (PA63). The PA63 oligomerizes to form a ring-shaped oligomer of seven or eight subunits (heptamer/octamer), which can then bind up to three molecules of EF and/or LF [79]. The PA-EF or PA-LF complex is then internalized via clathrin-mediated endocytosis. Upon endosomal acidification, PA undergoes a conformational change that facilitates pore formation, through which EF or LF translocate inside the cytoplasm [17]. The atomic structures of PA oligomer with LF and EF is accessible via PDB codes: 6PSN and 6UZB [79]. LF is a zinc-dependent metalloprotease. It acts and cleaves members of mitogen-activated protein kinase (MAPK) family, affecting MAPK pathways, crucial for cancer cell survival. In contrast, EF functions as a calmodulin-dependent adenylate cyclase and therefore acts by interfering with the cellular signaling pathway mediated by cyclic-adenosine monophosphate (cAMP), which eventually alters cellular physiology and contributes towards cell death [80].

The potential use of anthrax toxin as cancer therapeutics is due to its ability to target and kill cancer specific cells. Unlike conventional cancer therapies, the anthrax toxin-based agents can also be engineered so that they can have very high affinity and specificity towards cancer-specific receptors to generate quick and effective responses [81,82]. Introducing a double mutation (N682A/D683A) into domain 4 of PA compromised its native receptor-binding function, which can then be utilized to generate fusion proteins that target cancer-specific receptors [82]. One innovative approach exploits the tumor microenvironment, involving two proteases, namely matrix metalloproteinases (MMPs) and urokinase-type plasminogen activators (uPAs), which are usually overexpressed in cancer cells as compared to normal cells. Both the enzymes act synergistically towards cancer invasion and progression. Briefly, uPA activates plasmin, which degrades extracellular matrix (ECM) and also activates MMPs. These MMPs further break down ECM, thus enabling cancer cell invasion. By replacing PA’s native furin-cleavage site (aa 164–171) with sequences recognized by these tumor-associated proteases, engineered PA becomes selectively activated in cancer tissue. For example, in ovarian cancer, a modified PA (mPA) can bind to its receptor normally but is recognized and cleaved by MASPs, i.e., membrane-anchored serine proteases, which are overexpressed on the surface of cancer cells. This ensures the localized cytotoxic effect of the PA-LF, killing only cancer cells, keeping other cells unaffected [83].

Another targeted strategy was used to treat bladder cancer by fusing PA with epidermal growth factor (EGF) [84], leveraging the high expression of EGFRs in bladder tumor cells [85]. Briefly, this EGF-PA chimeric complex recognizes EGFR instead of native PA receptors, is proteolytically activated by furin protease to form oligomeric pore channel on the plasma membrane, and then recruits a fusion toxin LF_N_-DTA (N-terminal domain of LF fused to the catalytic domain of diphtheria toxin). This is followed by the internalization of the whole complex through receptor-mediated endocytosis. The acidification of the endosome results in conformational change in EGF-PA and promotes the translocation of LF_N_-DTA into the cytoplasm. This LF_N_-DTA then inactivates EF-2, halting protein synthesis and triggering apoptosis. This strategy was reported to be effective against human (T24), murine (MB49), and canine bladder cancer cell lines. This chimeric fusion protein was proposed to be a transformative therapy because it offers several advantages: the modified protein toxin is highly specific for the cells overexpressing EGFR; it is fast-acting (reducing treatment time from hours to minutes); and it is safe, requiring only nanomolar concentrations (LC100 ~2nM) for the complete killing of bladder cancer cells. This toxin complex is effective in the presence of human epidermal growth factor receptor-2 (HER2), suggesting that it is not affected by the HER2-mediated inhibition of EGFR internalization. Moreover, in vivo studies in mice and dogs have demonstrated a significant reduction in tumor burden without systemic toxicity [84].

Pancreatic cancer is one of the leading causes of deaths in men due to its late diagnosis, rapid progression and poor prognosis [13]. To target pancreatic cancer cells, an engineered immunotoxin was developed by fusing a scFv antibody to the C-terminus of a mPA. This specifically recognizes pancreatic cancer cell surface biomarkers, EGFR and carcinoembryonic antigen (CEA). Upon receptor binding, the complex facilitates the intracellular delivery of LF_N_-DTA into the cytosol which inhibits protein synthesis through the ADP ribosylation of EF-2 [86]. Additionally, the mPA-scFv fusion protein has also been utilized to deliver RRSP (Ras/Rap1-specific endopeptidase), which selectively cleaves and inactivates Ras and Rap1 proteins, thereby disrupting the Ras-ERK signaling pathway in pancreatic cancer cells [86]. IgG-engineered PA has also been utilized to deliver EF, LF_N_-DTA, and LF_N_-RRSP [87]. Since the Ras-ERK signaling pathway has been implicated in cancer development due to their role in the control of cell survival, growth, and differentiation, its targeted inhibition presents a promising therapeutic strategy in pancreatic cancer treatment [88].

Breast cancer continues to be the most common cancer affecting women globally [89]. In addition to ovarian and gastric carcinomas, the HER-2 receptor is frequently overexpressed in breast carcinoma [90]. The fusion proteins, mPA-ZHER2 (mPA fused to a HER2-specific affibody) and mPA-EGF, selectively target HER2-positive and EGF-positive cancer cells [91]. Both the modified toxins produced cytotoxic effects by recruiting LF_N_-DTA, thereby inhibiting protein synthesis and inducing apoptosis [91]. Breast tumors often exhibit the hyperactivation of MAPK/ERK signaling pathway and are therefore implicated in cancer progression [92]. El-Chami et al. investigated the effect of LeTx on breast cancer progression by targeting the MAPK/ERK pathway. LeTx significantly reduced cellular migration by decreasing cell motility and enhancing cell adhesion [93]. This enhanced cell adhesion was linked to the activation of the RhoA protein, a cytoskeletal regulator normally suppressed by Fos-related antigen 1 (FRA1), a downstream target of MAPK/ERK pathway [94]. The inactivation of ERK relieved FRA1-mediated inhibition, leading to the activation of RhoA-mediated cell adhesion. LeTx also effectively reduced cell invasion in MDA-MB-231 cells, a widely used model of triple-negative breast cancer, underlining its potential as a therapeutic strategy for breast cancer [93].

Gastric and colorectal cancers are among the leading causes of cancer-related mortality [13]. ANTXR1 or TEM8 is significantly overexpressed in various tumor cells [95,96,97] and silencing TEM8 leads to reduced cell proliferation, invasion, and metastasis, along with an increase in apoptosis, underscoring its role in the development of cancer development [95]. Multiple studies suggest that TEM8 could serve as a diagnostic marker and therapeutic target for multiple cancers, including gastric, colorectal, and lung cancers [97,98,99]. Engineered PA variants that selectively target TEM8 have shown strong potential as anti-cancer therapeutics [18]. Similarly, PA variants targeting related receptor CMG2 have also shown strong antitumor effects [100,101]. Thus, selective receptor targeting could improve therapeutic index and reduce off-target toxicity [18,78]. A summary of recent studies utilizing anthrax toxin variants is presented in Table 2.

While anthrax toxin-based agents are yet to advance to formal clinical trials for cancer therapy, promising results from preclinical research demonstrate their potential as anti-cancer therapeutics. Engineered variants of anthrax toxin have shown potent and selective anti-tumor activity in numerous models, both in vitro and in vivo. Figure 2 schematically describes the mechanisms of action of engineered anthrax toxin variants used in cancer therapy. These agents are well positioned to enter early human clinical trials, offering a highly targeted and mechanistically distinct option in the cancer therapy landscape. While PE and anthrax toxin have dominated recent developments in toxin-based cancer therapeutics, several other bacterial protein toxins, including the diphtheria toxin, *Clostridium* toxins, and botulinum toxin, have been explored in clinical or experimental settings, each offering unique mechanisms and targeting opportunities. Table 3 summarizes notable engineered toxin variants from other bacteria across a range of cancers.

## 4. Limitations and Future Directions

Bacterial protein toxins have diverse applications, including as therapeutic agents in oncology and neurology for disorders such as dystonia and chronic migraine. They also serve as key components in vaccines and are used in the delivery of therapeutic proteins. Additionally, *Bacillus thuringiensis* (Bt) toxins are widely utilized as environmentally safe bioinsecticides in agriculture, highlighting their broad utility across medicine, research, and industry [1,6,8]. Over the past decade, bacterial protein toxins, such as anthrax toxin, PE, diphtheria toxin, *Clostridium* enterotoxins, and Shiga toxin, have gained significant traction as precision-targeted anti-cancer agents. The genetic fusion of bacterial toxin domains with tumor-targeting ligands like mAbs, cytokines, or growth factors has emerged as a pivotal strategy to achieve greater target specificity (as described in Table 1, Table 2 and Table 3). Despite their therapeutic potential, bacterial toxin-based therapeutics face several key challenges. Immunogenicity remains a primary limitation, as these foreign proteins elicit NAbs responses that hinder repeated dosing and reduce therapeutic efficacy [14,18]. Advanced protein engineering strategies including systematic de-immunization through B-cell and T-cell epitope removal have shown promise in reducing immunogenicity while maintaining cytotoxic potency [14,120]. VLS represents another dose-limiting toxicity, caused by toxin binding to endothelial cells, though recent approaches involving immunosuppressive regimens have demonstrated improved tolerability [45,121]. Solid tumor penetration also presents an obstacle, as many PE-based immunotoxins struggle to achieve effective concentrations within large or poorly vascularized tumor masses [14]. PE immunotoxins typically utilize the classical retrograde transport pathway used by native toxins to reach cytosol; however, this process is variably efficient. Bypassing it through direct endosomal escape can overcome the limitations of the natural route. Poor endosomal escape remains a major limitation, as immunotoxins must exit the endosomal membrane and reach the cytoplasm before lysosomal degradation and inactivation. Inefficient escape necessitates higher doses to induce cell death, resulting in dose limitations and increased side effects [64]. Further, anthrax toxin-derived approaches face several critical issues on the path to clinical translation. Off-target toxicity remains a concern given the endogenous expression of anthrax toxin receptors (such as CMG2 and TEM8) on some normal tissues. Strictly restricting the activation of engineered anthrax toxins to the tumor microenvironment requires intricate protein engineering and is not always failproof. Because anthrax-based cancer therapeutics require administration as two separate proteins, achieving efficient complex formation and activity presents a significant challenge [18,122].

Current research focuses on overcoming these limitations through multiple innovative approaches [14]. Combination strategies with immunosuppressive agents like pentostatin and cyclophosphamide have successfully delayed anti-drug antibody formation, enabling multiple treatment cycles [123]. Novel targeting approaches including dual-protease activation systems and cell-surface receptor engineering are enhancing tumor selectivity in anthrax toxin-based targeting [18]. Tumor-selective PA variants have been engineered and further modified at LF-binding sites to create intermolecular complementing pairs. This strategy allows the precise activation of the anthrax toxin complex specifically at tumor sites [124]. The development of SO1861 enhancement technology represents a breakthrough in overcoming endosomal escape limitations that have historically restricted immunotoxin efficacy [64]. Subcutaneous delivery approaches have emerged as promising alternatives to traditional intravenous administration, offering improved pharmacokinetic profiles and enhanced tumor targeting [16]. Future directions include the humanization of antibody and protein-based agents through the grafting of complementarity-determining regions (CDRs) onto a human antibody scaffold and next-generation computational design to reduce immunogenicity [125]. Targeted nanoparticle systems incorporating bacterial toxins have demonstrated improved tumor penetration and reduced systemic toxicity compared to free toxin formulations, addressing longstanding challenges in solid tumor applications [126]. The emergence of living bacterial therapeutics represents a revolutionary approach to cancer treatment that use live, genetically engineered bacteria to produce and deliver bacterial toxins directly within tumor microenvironments [127]. CRISPR/Cas9 technology has further enhanced the precision of bacterial therapeutic engineering, enabling the modification of *Clostridium novyi*-NT and other anaerobic bacteria to incorporate tumor-targeting mechanisms [118].

Cancer cells can develop resistance to bacterial toxin-based therapeutics through multiple sophisticated mechanisms that must be understood and addressed for successful clinical application. Antigen loss or downregulation represents one of the most common resistance pathways. Cancer cells may develop mutations affecting clathrin-mediated endocytosis, receptor recycling pathways, or endosomal acidification processes that are critical for toxin activation and translocation. Target protein modifications are another way wherein cancer cells alter the target proteins themselves to reduce toxin binding or catalytic activity [74]. In this regard, combination strategies have emerged as effective approaches to overcome resistance and enhance therapeutic efficacy. The integration of bacterial toxin immunotoxins with immune checkpoint inhibitors has shown remarkable synergistic effects [14]. Additionally, proteasome inhibitors such as bortezomib have been shown to reduce preexisting NAbs to immunotoxins in mice by depleting antibody-producing plasma cells [128]. Furthermore, combining immuotoxins with small molecule inhibitors like ABT-737 enhances intracellular immunotoxin accumulation by increasing toxin delivery inside the cell and promoting apoptotic cell death [74,129].

Understanding the molecular mechanisms through which bacterial toxins act is crucial for harnessing their potential as tools to modulate specific cellular pathways. By converting their naturally harmful effects into beneficial outcomes, these toxins can be repurposed for therapeutic applications. Continued research into toxin–host interactions will enhance our ability to develop targeted treatment strategies and improve clinical outcomes.

The clinical approval of PE-based moxetumomab pasudotox (Lumoxiti) for hairy cell leukemia and diphtheria toxin-based denileukin diftitox (Ontak) for cutaneous T-cell lymphoma marked a significant milestone in the development of bacterial toxin-based cancer therapies [12]. However, their subsequent withdrawal from the market, largely due to commercial, manufacturing, and logistical challenges, reflect the real-world challenges of translating biotherapeutics into sustainable clinical practice. Ongoing advances in protein engineering, delivery technologies, and combination strategies hold promise for overcoming these hurdles, paving the way for more effective, accessible, and durable bacterial toxin-derived treatments in oncology.

## 5. Conclusions

The transformation of bacterial protein toxins from virulence factors into sophisticated cancer therapeutics exemplifies a remarkable example of translational medicine in modern oncology. The clinical approval of PE-based moxetumomab pasudotox has validated bacterial toxins as a viable therapeutic class, while ongoing developments in anthrax toxin engineering demonstrate the continued potential for innovation in this field. Recent advances in protein engineering, immunology, and combination therapy strategies position bacterial toxin-based cancer therapeutics at the forefront of precision oncology, offering new hope for treating previously intractable cancers.

## Figures and Tables

**Table 1:** *Pseudomonas* exotoxin A (PE)-based therapeutic trials.

Agent	Target	Cancer Type/s	Phase and Status	Key Findings	Reference/s
**Moxetumomab pasudotox (HA22,** **PE38-based)** **Moxetumomab +** **rituximab**	CD22	Relapsed/refractory hairy cell leukemia (HCL)	Phase III (NCT01829711) FDA-approved 2018	41% CR in HCL adults, most MRD-negative	[52]
		Relapsed/refractory childhood ALL	Phase I (NCT00659425)	32% ORR in pediatric ALL (23% CR; 5 MRD-neg)	[53,54]
		HCL	Phase I (NCT03805932)	78% CR and 72% MRD-free CR with reduced immunogenicity	[55]
**LMB-100** **(anti-mesothelin** **Fab-PE24)**	Mesothelin	Mesothelioma, pancreatic adenocarcinoma	Phase I (NCT02798536, NCT02317419)	Renal toxicity; short half-life (53 min); reduced efficacy. Synergistic effects with checkpoint inhibitor, pembrolizumab	[56]
**LMB-100 + nab-** **paclitaxel**	Mesothelin	Advanced pancreatic cancer	Phase I/II (NCT02810418)	Significant antitumor activity in 40% patients; severe CLS; combination discontinued	[57]
**LMB-100 +** **Tofacitinib**	Mesothelin/ JAK	Solid tumors	Phase I (NCT04034238)	Study terminated early due to pericarditis in two patients, ADA prevention shown	[58]
**SS1(dsFV)PE38 +** **pemetrexed/** **cisplatin**	Mesothelin	Mesothelioma	Phase I (NCT01445392)	SS1P + chemotherapy achieved 60% ORR; 77% at MTD; NAbs formed in 90% after one cycle	[59]
**BL22 (RFB4-dsFv-** **PE38)**	CD22	Relapsed/refractory hairy cell leukemia	Phase II (NCT00074048)	25% CR after one cycle (47% after retreatment); 64% CR if spleen <200 mm; HUS in 6%	[60,61]
**LMB-2 [anti-** **Tac(Fv)-PE38] +** **chemotherapy**	CD25 (IL-2R alpha chain, Tac antigen)	Adult T-cell leukemia/ lymphoma	Phase I/II (NCT00924170)	60% CR, particularly in leukemia; chemotherapy reduced ADAs	[62,63]
**hD7-1(VL-VH)-** **PE24mut**	PSMA	Prostate cancer	Preclinical (in vivo)	REDLK deletion + endosomal escape enhancer SO1861 significantly increases cytotoxicity of PE	[64]
**OVB3-PE**	OVCAR antigens	Ovarian cancer	Phase I	No clinically significant antitumor activity; CNS expression caused lethal DLT	[65]
**FRP-PE40**	HER2/ ErbB2	Metastatic breast, prostate, head/neck, lung carcinoma	Phase I	Limited efficacy due to tumor accessibility; immunogenicity by week 2; no CR; stable disease in 11% patients	[66]
**SGN-10 (BR96** **sFv-PE40)**	Le^Y^ (Lewis Y) antigen	Colon, pancreatic carcinoma	Phase I	No antitumor response; rapid immunological clearance, limiting efficacy despite stable disease in 31% patients	[67]
**MOC31PE**	EpCAM (CD326)	Colorectal, peritoneal carcinomatosis	Phase I/II (NCT02219893)	Good PK/tolerability; safe IP dosing up to 10 μg/kg; no radiological complete/partial response observed; 36% had stable disease	[68]
**Vicinium** **(VB4-845 or** **Oportuzumab monatox)**	EpCAM	BCG-refractory bladder cancer	Phase II (NCT00462488, NCT02449239)	16% disease-free at 24 months; well tolerated	[69]
**D2C7-IT (D2C7-** **(scdsFv)-PE38KDEL)**	EGFR/ EGFRvIII	Glioblastoma	Phase I/II (NCT02303678); Phase I with anti- CD40 (NCT04547777)	Partial responses; optimal dosage established; CD40 combination shows promise	[70]
**Cintredekin besudotox (PE38QQR)**	IL-13R	Glioblastoma	Phase III (NCT00024570)	Phase I/II showed efficacy but Phase III terminated; thromboembolic toxicity	[71,72,73]

Abbreviations: PE, *Pseudomonas* exotoxin A; PE38, a 38 kDa truncated PE fragment used in immunotoxins; PE24, a 24 kDa truncated and de-immunized fragment of PE; scFv, single-chain variable fragment; HCL, hairy cell leukemia; CR, complete remission; Fab, fragment antigen-binding; MRD, minimal residual disease; ORR, objective response rate; CLS, capillary leak syndrome; ALL, acute lymphoblastic leukemia; MTD, maximum tolerated dose; ADA, antidrug antibodies; NAbs, neutralizing antibodies; EpCAM, epithelial cell adhesion molecule, PSMA, prostate-specific membrane antigen; DLT, dose-limiting toxicity; PK, pharmacokinetics; IP, intraperitoneal; HER2, human epidermal growth factor receptor 2; BCG, Bacillus Calmette–Guerin; EGFR, epidermal growth factor receptor; JAK, Janus kinase; FDA, Food and Drug Administration, USA.

**Table 2:** Anthrax toxin-based therapeutic trials.

Agent	Target/ Activation	Cancer Type/s	Phase and Status	Key Findings	Reference
**PAS** **(prostasin-activated PA)**	MASPs (membrane-anchored serine proteases)	Ovarian cancer	Preclinical (in vivo)	Highly specific cytotoxicity; only binds MASP-overexpressing cells	[83]
**EGF-PA′**	EGFR	HER2 +/− bladder cancer	Preclinical (in vivo)	Drives internalization via PA oligomerization; overcomes EGFR mutations and HER2-mediated inhibition of endocytosis	[84]
**PA-L1/LF**	MMP activation	Melanoma (BRAF V600E)	Preclinical (in vivo)	MEK inactivation confirmed indicating successful delivery of the active toxin to the MAPK pathway; BRAF-mutant cells highly susceptible to both PA/LF and PA-L1/LF	[102]
**LFnCdtB**	TEM8/CMG2 (ANTXR1/2)	Melanoma, colon, lung cancer	Preclinical (in vivo)	*H. ducreyi* CdtB fused to N-terminal 255 aa of *B. anthracis* LFn was engineered. PA-L1+LFnCdtB cured 8/10 melanoma mice; no toxicity vs. severe toxicity with wild-type PA	[103]
**CLPA (Bismaleimide cross-linked** **PA variants)** **[PA-L1 and** **PA-U2]**	TEM8/CMG2 (ANTXR1/2)	Melanoma, colon, lung cancer	Preclinical (in vivo)	Functional octamers; highly specific anti-angiogenic activity of this dual-protease-activated toxin; improved manufacturability	[104]
**PA-U2 + LF**	uPA activation	Fibrosarcoma, melanoma, Lewis lung carcinoma	Preclinical (in vivo)	Furin cleavage site in PA is replaced by an artificial uPA substrate sequence; eliminated systemic toxicity, while maintaining tumor-specific cytotoxicity	[105]
**PA-L1-I207R** **+ PA-U2-** **R200A**	MMP and uPA dual activation via TEM8/ ANTXR1	Lewis lung carcinoma	Preclinical (in vivo)	Dose-dependent anti-tumor response; cyclophosphamide prevented NAbs	[106]
**PA-R659S/** **M662R** **variants**	TEM8/CMG2 receptor selectivity	Human tumor cells	Preclinical (in vitro)	Enhanced CMG2 selectivity; reduced toxicity in CMG2-deficient mice	[100]

Abbreviations: PA, protective antigen; LFn, anthrax toxin lethal factor; TEM8, tumor endothelial marker 8; EGFR, epidermal growth factor receptor; MMP, matrix metalloproteinases; MAPK, mitogen-activated protein kinase; MEK, MAPK kinase; BRAF, B-raf proto-oncogene, serine/threonine kinase; HER2, human epidermal growth factor receptor 2; CMG2, capillary morphogenesis protein 2; ANTXR1/2, anthrax toxin receptors 1 and 2; PA-L1, matrix metalloproteinase-activated PA; PA-U2, urokinase plasminogen activator-activated PA; CdtB, cytolethal distending toxin; NAbs, neutralizing antibodies.

**Table 3:** Other bacterial toxin-based therapeutic trials.

Agent	Target	Cancer Type/s	Phase and Status	Key Findings	Reference/s
**Denileukin** **diftitox** **(DAB389-IL-2)** **diphtheria** **toxin-based**	IL-2R	Melanoma, CTCL, CLL, ovarian cancer	FDA-Approved 2008 (CTCL); Phase II Melanoma (NCT00299689)	Significant activity in Stage IV melanoma: 1-year OS 40% vs. historical 25.5%; Treg depletion mechanism	[107]
**Tagraxofusp** **SL-401** **(DT388-IL-3)**	IL-3RA (CD123)	BPDCN, AML	FDA-Approved 2018 (BPDCN) Phase I/II (NCT00397579)	72% complete or clinical response rate in BPDCN; 90% ORR; 52% 2-year survival; side effects included elevated liver enzymes, hypoalbuminemia, edema, and thrombocytopenia.	[108,109]
**Tf-CRM107** **(DT-Tf)**	Transferrin receptor	GBM	Phase I/II (NCT00052624)	Promising tumor responses; neurotoxicity due to vascular Tf receptor expression	[110]
**DAB389EGF** **(DT-based)**	EGFR	GBM, bladder, breast cancer	Preclinical	Significant tumor reduction in orthotopic mice models; intravesical delivery effective	[111]
**DT2219ARL** **(DT-based)**	CD19 and CD22	B-cell malignancies	Phase I/II (NCT00889408)	Superior efficacy vs. monospecific variants; long-term tumor-free survival in SCID models	[112]
**MT-3724** **(anti-CD20** **scFv fused with** **Shiga-like toxin A subunit)**	CD20	Relapsed/ refractory B-cell NHL	Phase Ia/b (NCT02302381)	21.7% ORR overall; 41.7% ORR in rituximab-naïve DLBCL	[113]
***Clostridium*** ***septicum*** **α-toxin**	GPI- anchored proteins	Breast cancer	Preclinical	α-toxin gene expressed in *E. coli* showed anti-cancer effects on MCF-7 breast cancer cells; significantly reduced tumor size in animal models.	[114]
**Botulinum toxin** **A (BTX)** **(*Clostridium*** ***botulinum*)**	TRPM2	Glioblastoma, neuroblastoma	Preclinical	Induced cell death via mitochondrial ROS and TRPM2 signaling	[115]
***Clostridium perfringens*** **enterotoxin (CPE)**	CLDN4	Pancreatic cancer	Preclinical	CPE-conjugated polysialic acid nanoparticles enhanced targeting and enhanced permeability and retention effect	[116]
***Clostridium*** ***difficile* toxin B** **(cdtB)**	C-terminal CROPs/MBM regions	Breast cancer	Preclinical	Suppressed Bcl-2, decreased C-erbB-2 and Cox-2 expression in breast cancer mouse model	[117]
***Clostridium*** ***novyi*-NT +** **RGD peptide** **modification**	αvβ3 integrin	Pancreatic cancer	Preclinical/ Phase 1 (NCT01924689)	Enhanced tumor localization with smaller dose via CRISPR/Cas9 modification	[118,119]

Abbreviations: DT, diphtheria toxin; OS, overall survival; CTCL, cutaneous T-cell lymphoma; CLL, chronic lymphocytic leukemia; AML, acute myeloid leukemia; ORR, objective response rate; Tf, transferrin; DLBCL, diffuse large B-cell lymphoma; MCF-7, Michigan Cancer Foundation-7 developed human breast cancer cell line; BPDCN, blastic plasmacytoid dendritic cell neoplasm; ROS, reactive oxygen species; CLDN4, claudin-4; CROPs, combined repetitive oligopeptides; MBM, membrane binding motif; RGD peptide, arginine-glycine-aspartic acid peptide; GBM, glioblastoma multiforme; FDA, Food and Drug Administration, USA; TRPM2, Transient receptor potential cation channel, subfamily M, member 2.

## Data Availability

No new data were created or analyzed in this study.

## References

[1] Popoff M.R. (2024). Overview of Bacterial Protein Toxins from Pathogenic Bacteria: Mode of Action and Insights into Evolution. Toxins.

[2] Xiao Y., Yan Z., Ren F., Tan Y. (2025). Bacterial Exotoxins in Medicine: Potential Value and Perspectives. Int. J. Med. Sci..

[3] Aepfelbacher M., Aktories K., Just I. (2000). Bacterial Protein Toxins. Handbook of Experimental Pharmacology.

[4] Wilson J.W., Rolland A.D., Klausen G.M., Prell J.S. (2019). Ion Mobility-Mass Spectrometry Reveals That α-Hemolysin from *Staphylococcus Aureus* Simultaneously Forms Hexameric and Heptameric Complexes in Detergent Micelle Solutions. Anal. Chem..

[5] Oda M., Terao Y., Sakurai J., Nagahama M. (2015). Membrane-Binding Mechanism of *Clostridium Perfringens* Alpha-Toxin. Toxins.

[6] Márquez-López A., Fanarraga M.L. (2023). AB Toxins as High-Affinity Ligands for Cell Targeting in Cancer Therapy. Int. J. Mol. Sci..

[7] Lemichez E., Barbieri J.T. (2013). General Aspects and Recent Advances on Bacterial Protein Toxins. Cold Spring Harb. Perspect. Med..

[8] Fabbri A., Rosadi F., Ballan G., Del Brocco A., Travaglione S., Loizzo S., Fiorentini C., Atta-ur-Rahman, Reitz A.B., Choudhary I., Wang J. (2015). Bacterial Protein Toxins: Current and Potential Clinical Use. Frontiers in Medicinal Chemistry.

[9] Carlson R.D., Flickinger J.C., Snook A.E. (2020). Talkin’ Toxins: From Coley’s to Modern Cancer Immunotherapy. Toxins.

[10] Shapira A., Benhar I. (2010). Toxin-Based Therapeutic Approaches. Toxins.

[11] Hymes K. (2008). Denileukin Diftitox for the Treatment of Cutaneous T-Cell Lymphoma. Biol. Targets Ther..

[12] Khoshnood S., Fathizadeh H., Neamati F., Negahdari B., Baindara P., Abdullah M.A., Haddadi M.H. (2022). Bacteria-Derived Chimeric Toxins as Potential Anticancer Agents. Front. Oncol..

[13] Bray F., Laversanne M., Sung H., Ferlay J., Siegel R.L., Soerjomataram I., Jemal A. (2024). Global Cancer Statistics 2022: GLOBOCAN Estimates of Incidence and Mortality Worldwide for 36 Cancers in 185 Countries. CA A Cancer J. Clin..

[14] Morgan R.N., Saleh S.E., Farrag H.A., Aboshanab K.M. (2023). New Insights on *Pseudomonas aeruginosa* Exotoxin A-Based Immunotoxins in Targeted Cancer Therapeutic Delivery. Ther. Deliv..

[15] Havaei S.M., Aucoin M.G., Jahanian-Najafabadi A. (2021). Pseudomonas Exotoxin-Based Immunotoxins: Over Three Decades of Efforts on Targeting Cancer Cells with the Toxin. Front. Oncol..

[16] Pang Z., Gu M.-D., Tang T. (2022). *Pseudomonas aeruginosa* in Cancer Therapy: Current Knowledge, Challenges and Future Perspectives. Front. Oncol..

[17] Friebe S., Van Der Goot F., Bürgi J. (2016). The Ins and Outs of Anthrax Toxin. Toxins.

[18] Bachran C., Leppla S. (2016). Tumor Targeting and Drug Delivery by Anthrax Toxin. Toxins.

[19] Yin L., Thaker H. (2023). Cancer Drug Delivery Systems Using Bacterial Toxin Translocation Mechanisms. Bioengineering.

[20] Qin S., Xiao W., Zhou C., Pu Q., Deng X., Lan L., Liang H., Song X., Wu M. (2022). *Pseudomonas aeruginosa*: Pathogenesis, Virulence Factors, Antibiotic Resistance, Interaction with Host, Technology Advances and Emerging Therapeutics. Signal Transduct. Target. Ther..

[21] Odumosu O., Nicholas D., Yano H., Langridge W. (2010). AB Toxins: A Paradigm Switch from Deadly to Desirable. Toxins.

[22] Wedekind J.E., Trame C.B., Dorywalska M., Koehl P., Raschke T.M., McKee M., FitzGerald D., Collier R.J., McKay D.B. (2001). Refined Crystallographic Structure of *Pseudomonas aeruginosa* Exotoxin A and Its Implications for the Molecular Mechanism of Toxicity 1 1Edited by D. Rees. J. Mol. Biol..

[23] Wolf P., Elsässer-Beile U. (2009). Pseudomonas Exotoxin A: From Virulence Factor to Anti-Cancer Agent. Int. J. Med. Microbiol..

[24] Kounnas M.Z., Morris R.E., Thompson M.R., FitzGerald D.J., Strickland D.K., Saelinger C.B. (1992). The Alpha 2-Macroglobulin Receptor/Low Density Lipoprotein Receptor-Related Protein Binds and Internalizes Pseudomonas Exotoxin A. J. Biol. Chem..

[25] Ogata M., Fryling C.M., Pastan I., FitzGerald D.J. (1992). Cell-Mediated Cleavage of Pseudomonas Exotoxin between Arg279 and Gly280 Generates the Enzymatically Active Fragment Which Translocates to the Cytosol. J. Biol. Chem..

[26] McKee M.L., FitzGerald D.J. (1999). Reduction of Furin-Nicked Pseudomonas Exotoxin A: An Unfolding Story. Biochemistry.

[27] Hessler J.L., Kreitman R.J. (1997). An Early Step in Pseudomonas Exotoxin Action Is Removal of the Terminal Lysine Residue, Which Allows Binding to the KDEL Receptor. Biochemistry.

[28] Kreitman R.J., Pastan I. (1995). Importance of the Glutamate Residue of KDEL in Increasing the Cytotoxicity of *Pseudomonas* Exotoxin Derivatives and for Increased Binding to the KDEL Receptor. Biochem. J..

[29] Jackson M.E., Simpson J.C., Girod A., Pepperkok R., Roberts L.M., Lord J.M. (1999). The KDEL Retrieval System Is Exploited by *Pseudomonas* Exotoxin A, but Not by Shiga-like Toxin-1, during Retrograde Transport from the Golgi Complex to the Endoplasmic Reticulum. J. Cell Sci..

[30] Hazes B., Read R.J. (1997). Accumulating Evidence Suggests That Several AB-Toxins Subvert the Endoplasmic Reticulum-Associated Protein Degradation Pathway to Enter Target Cells. Biochemistry.

[31] Koopmann J.-O., Albring J., Hüter E., Bulbuc N., Spee P., Neefjes J., Hämmerling G.J., Momburg F. (2000). Export of Antigenic Peptides from the Endoplasmic Reticulum Intersects with Retrograde Protein Translocation through the Sec61p Channel. Immunity.

[32] Iglewski B.H., Liu P.V., Kabat D. (1977). Mechanism of Action of *Pseudomonas aeruginosa* Exotoxin Aiadenosine Diphosphate-Ribosylation of Mammalian Elongation Factor 2 in Vitro and in Vivo. Infect. Immun..

[33] Jørgensen R., Merrill A.R., Yates S.P., Marquez V.E., Schwan A.L., Boesen T., Andersen G.R. (2005). Exotoxin A–eEF2 Complex Structure Indicates ADP Ribosylation by Ribosome Mimicry. Nature.

[34] Yates S.P., Merrill A.R. (2004). Elucidation of Eukaryotic Elongation Factor-2 Contact Sites within the Catalytic Domain of *Pseudomonas aeruginosa* Exotoxin A. Biochem. J..

[35] Li M., Dyda F., Benhar I., Pastan I., Davies D.R. (1996). Crystal Structure of the Catalytic Domain of Pseudomonas Exotoxin A Complexed with a Nicotinamide Adenine Dinucleotide Analog: Implications for the Activation Process and for ADP Ribosylation. Proc. Natl. Acad. Sci. USA.

[36] Armstrong S., Li J.-H., Zhang J., Rod Merrill A. (2002). Characterization of Competitive Inhibitors for the Transferase Activity of *Pseudomonas aeruginosa* Exotoxin A. J. Enzyme Inhib. Med. Chem..

[37] Ortiz P.A. (2005). Dominant-Negative Mutant Phenotypes and the Regulation of Translation Elongation Factor 2 Levels in Yeast. Nucleic Acids Res..

[38] Proud C.G. (1994). Peptide-Chain Elongation in Eukaryotes. Mol. Biol. Rep..

[39] Chang J.-H., Kwon H.-Y. (2007). Expression of 14-3-3δ, Cdc2 and Cyclin B Proteins Related to Exotoxin A-Induced Apoptosis in HeLa S3 Cells. Int. Immunopharmacol..

[40] Antignani A., Segal D., Simon N., Kreitman R.J., Huang D., FitzGerald D.J. (2017). Essential Role for Bim in Mediating the Apoptotic and Antitumor Activities of Immunotoxins. Oncogene.

[41] Pai L.H. (1993). Immunotoxin Therapy for Cancer. JAMA.

[42] Kondo T., FitzGerald D., Chaudhary V.K., Adhya S., Pastan I. (1988). Activity of Immunotoxins Constructed with Modified Pseudomonas Exotoxin A Lacking the Cell Recognition Domain. J. Biol. Chem..

[43] Pai L.H., Wittes R., Setser A., Willingham M.C., Pastan I. (1996). Treatment of Advanced Solid Tumors with Immunotoxin LMB–1: An Antibody Linked to Pseudomonas Exotoxin. Nat. Med..

[44] Pai L.H., Pastan I., Frankel A.E. (1998). Clinical Trials with *Pseudomonas* Exotoxin Immunotoxins. Clinical Applications of Immunotoxins.

[45] Siegall C.B., Liggitt D., Chace D., Mixan B., Sugai J., Davidson T., Steinitz M. (1997). Characterization of Vascular Leak Syndrome Induced by the Toxin Component of Pseudomonas Exotoxin-Based Immunotoxins and Its Potential Inhibition with Nonsteroidal Anti-Inflammatory Drugs. Clin. Cancer Res. Off. J. Am. Assoc. Cancer Res..

[46] Chowdhury P.S., Viner J.L., Beers R., Pastan I. (1998). Isolation of a High-Affinity Stable Single-Chain Fv Specific for Mesothelin from DNA-Immunized Mice by Phage Display and Construction of a Recombinant Immunotoxin with Anti-Tumor Activity. Proc. Natl. Acad. Sci. USA.

[47] Debinski W., Pastan I. (1994). An Immunotoxin with Increased Activity and Homogeneity Produced by Reducing the Number of Lysine Residues in Recombinant Pseudomonas Exotoxin. Bioconjugate Chem..

[48] Iwamoto F.M., Lamborn K.R., Robins H.I., Mehta M.P., Chang S.M., Butowski N.A., DeAngelis L.M., Abrey L.E., Zhang W.-T., Prados M.D. (2010). Phase II Trial of Pazopanib (GW786034), an Oral Multi-Targeted Angiogenesis Inhibitor, for Adults with Recurrent Glioblastoma (North American Brain Tumor Consortium Study 06-02). Neuro-Oncology.

[49] Theuer C.P., Fitzgerald D.J., Pastan I. (1993). A Recombinant Form of Pseudomonas Exotoxin a Containing Transforming Growth Factor Alpha Near Its Carboxyl Terminus for the Treatment of Bladder Cancer. J. Urol..

[50] Bauss F., Lechmann M., Krippendorff B.-F., Staack R., Herting F., Festag M., Imhof-Jung S., Hesse F., Pompiati M., Kollmorgen G. (2016). Characterization of a Re-engineered, Mesothelin-targeted *Pseudomonas* Exotoxin Fusion Protein for Lung Cancer Therapy. Mol. Oncol..

[51] Goleij Z., Mahmoodzadeh Hosseini H., Sedighian H., Behzadi E., Halabian R., Sorouri R., Imani Fooladi A.A. (2020). Breast Cancer Targeted/Therapeutic with Double and Triple Fusion Immunotoxins. J. Steroid Biochem. Mol. Biol..

[52] The Study 1053 investigators, Kreitman R.J., Dearden C., Zinzani P.L., Delgado J., Robak T., Le Coutre P.D., Gjertsen B.T., Troussard X., Roboz G.J. (2021). Moxetumomab Pasudotox in Heavily Pre-Treated Patients with Relapsed/Refractory Hairy Cell Leukemia (HCL): Long-Term Follow-up from the Pivotal Trial. J. Hematol. Oncol..

[53] Wayne A.S., Shah N.N., Bhojwani D., Silverman L.B., Whitlock J.A., Stetler-Stevenson M., Sun W., Liang M., Yang J., Kreitman R.J. (2017). Phase 1 Study of the Anti-CD22 Immunotoxin Moxetumomab Pasudotox for Childhood Acute Lymphoblastic Leukemia. Blood.

[54] Shah N.N., Bhojwani D., August K., Baruchel A., Bertrand Y., Boklan J., Dalla-Pozza L., Dennis R., Hijiya N., Locatelli F. (2020). Results from an International Phase 2 Study of the anti-CD22 Immunotoxin Moxetumomab Pasudotox in Relapsed or Refractory Childhood B-lineage Acute Lymphoblastic Leukemia. Pediatr. Blood Cancer.

[55] Kreitman R.J., Yuan C., Wang H.-W., Zhou H., Raffeld M., Xi L., Arons E., Feurtado J., James-Echenique L., Steinberg S.M. (2024). Effect of Rituximab on Remissions without Minimal Residual Disease and Immunogenicity in Patients with Relapsed/Refractory Hairy Cell Leukemia Receiving Moxetumomab Pasudotox. J. Clin. Oncol..

[56] Hassan R., Alewine C., Mian I., Spreafico A., Siu L.L., Gomez-Roca C., Delord J., Italiano A., Lassen U., Soria J. (2020). Phase 1 Study of the Immunotoxin LMB-100 in Patients with Mesothelioma and Other Solid Tumors Expressing Mesothelin. Cancer.

[57] Alewine C., Ahmad M., Peer C.J., Hu Z.I., Lee M.-J., Yuno A., Kindrick J.D., Thomas A., Steinberg S.M., Trepel J.B. (2020). Phase I/II Study of the Mesothelin-Targeted Immunotoxin LMB-100 with Nab-Paclitaxel for Patients with Advanced Pancreatic Adenocarcinoma. Clin. Cancer Res..

[58] Skorupan N., Peer C.J., Zhang X., Choo-Wosoba H., Ahmad M.I., Lee M.-J., Rastogi S., Sato N., Yu Y., Pegna G.J. (2024). Tofacitinib to Prevent Anti-Drug Antibody Formation against LMB-100 Immunotoxin in Patients with Advanced Mesothelin-Expressing Cancers. Front. Oncol..

[59] Hassan R., Sharon E., Thomas A., Zhang J., Ling A., Miettinen M., Kreitman R.J., Steinberg S.M., Hollevoet K., Pastan I. (2014). Phase 1 Study of the Antimesothelin Immunotoxin SS1P in Combination with Pemetrexed and Cisplatin for Front-line Therapy of Pleural Mesothelioma and Correlation of Tumor Response with Serum Mesothelin, Megakaryocyte Potentiating Factor, and Cancer Antigen 125. Cancer.

[60] Kreitman R.J., Stetler-Stevenson M., Margulies I., Noel P., FitzGerald D.J.P., Wilson W.H., Pastan I. (2009). Phase II Trial of Recombinant Immunotoxin RFB4(dsFv)-PE38 (BL22) in Patients with Hairy Cell Leukemia. J. Clin. Oncol..

[61] Wayne A.S., Kreitman R.J., Findley H.W., Lew G., Delbrook C., Steinberg S.M., Stetler-Stevenson M., FitzGerald D.J., Pastan I. (2010). Anti-CD22 Immunotoxin RFB4(dsFv)-PE38 (BL22) for CD22-Positive Hematologic Malignancies of Childhood: Preclinical Studies and Phase I Clinical Trial. Clin. Cancer Res..

[62] Kreitman R.J., Stetler-Stevenson M., Jaffe E.S., Conlon K.C., Steinberg S.M., Wilson W., Waldmann T.A., Pastan I. (2016). Complete Remissions of Adult T-Cell Leukemia with Anti-CD25 Recombinant Immunotoxin LMB-2 and Chemotherapy to Block Immunogenicity. Clin. Cancer Res..

[63] Kreitman R.J., Wilson W.H., White J.D., Stetler-Stevenson M., Jaffe E.S., Giardina S., Waldmann T.A., Pastan I. (2000). Phase I Trial of Recombinant Immunotoxin Anti-Tac(Fv)-PE38 (LMB-2) in Patients with Hematologic Malignancies. J. Clin. Oncol..

[64] Masilamani A.P., Huber N., Nagl C., Dettmer-Monaco V., Monaco G., Wolf I., Schultze-Seemann S., Taromi S., Gratzke C., Fuchs H. (2023). Enhanced Cytotoxicity of a Pseudomonas Exotoxin A Based Immunotoxin against Prostate Cancer by Addition of the Endosomal Escape Enhancer SO1861. Front. Pharmacol..

[65] Pai B.L.H., Bookman M.A., Ozols R.F., Young R.C., Smith J.W., Longo D.L., Gould B., Frankel A., McClay E.F., Howell S. (1991). Clinical Evaluation of Intraperitoneal Pseudomonas Exotoxin Immunoconjugate OVB3-PE in Patients with Ovarian Cancer. J. Clin. Oncol..

[66] Von Minckwitz G., Harder S., Hövelmann S., Jäger E., Al-Batran S.-E., Loibl S., Atmaca A., Cimpoiasu C., Neumann A., Abera A. (2005). Phase I Clinical Study of the Recombinant Antibody Toxin scFv(FRP5)-ETA Specific for the ErbB2/HER2 Receptor in Patients with Advanced Solid Malignomas. Breast Cancer Res..

[67] Posey J.A., Khazaeli M.B., Bookman M.A., Nowrouzi A., Grizzle W.E., Thornton J., Carey D.E., Lorenz J.M., Sing A.P., Siegall C.B. (2002). A Phase I Trial of the Single-Chain Immunotoxin SGN-10 (BR96 sFv-PE40) in Patients with Advanced Solid Tumors. Clin. Cancer Res. Off. J. Am. Assoc. Cancer Res..

[68] Andersson Y., Engebraaten O., Juell S., Aamdal S., Brunsvig P., Fodstad Ø., Dueland S. (2015). Phase I Trial of EpCAM-Targeting Immunotoxin MOC31PE, Alone and in Combination with Cyclosporin. Br. J. Cancer.

[69] Kowalski M., Guindon J., Brazas L., Moore C., Entwistle J., Cizeau J., Jewett M.A.S., MacDonald G.C. (2012). A Phase II Study of Oportuzumab Monatox: An Immunotoxin Therapy for Patients with Noninvasive Urothelial Carcinoma In Situ Previously Treated with Bacillus Calmette-Guérin. J. Urol..

[70] Parker S., McDowall C., Sanchez-Perez L., Osorio C., Duncker P.C., Briley A., Swartz A.M., Herndon J.E., Yu Y.-R.A., McLendon R.E. (2023). Immunotoxin-αCD40 Therapy Activates Innate and Adaptive Immunity and Generates a Durable Antitumor Response in Glioblastoma Models. Sci. Transl. Med..

[71] Bhardwaj R., Suzuki A., Leland P., Joshi B.H., Puri R.K. (2018). Identification of a Novel Role of IL-13Rα2 in Human Glioblastoma Multiforme: Interleukin-13 Mediates Signal Transduction through AP-1 Pathway. J. Transl. Med..

[72] Kunwar S., Chang S., Westphal M., Vogelbaum M., Sampson J., Barnett G., Shaffrey M., Ram Z., Piepmeier J., Prados M. (2010). Phase III Randomized Trial of CED of IL13-PE38QQR vs Gliadel Wafers for Recurrent Glioblastoma. Neuro-Oncology.

[73] Heiss J.D., Jamshidi A., Shah S., Martin S., Wolters P.L., Argersinger D.P., Warren K.E., Lonser R.R. (2019). Phase I Trial of Convection-Enhanced Delivery of IL13-Pseudomonas Toxin in Children with Diffuse Intrinsic Pontine Glioma. J. Neurosurg. Pediatr..

[74] Dieffenbach M., Pastan I. (2020). Mechanisms of Resistance to Immunotoxins Containing Pseudomonas Exotoxin A in Cancer Therapy. Biomolecules.

[75] St Croix B., Rago C., Velculescu V., Traverso G., Romans K.E., Montgomery E., Lal A., Riggins G.J., Lengauer C., Vogelstein B. (2000). Genes Expressed in Human Tumor Endothelium. Science.

[76] Carson-Walter E.B., Watkins D.N., Nanda A., Vogelstein B., Kinzler K.W., St Croix B. (2001). Cell Surface Tumor Endothelial Markers Are Conserved in Mice and Humans. Cancer Res..

[77] Reeves C.V., Dufraine J., Young J.A.T., Kitajewski J. (2010). Anthrax Toxin Receptor 2 Is Expressed in Murine and Tumor Vasculature and Functions in Endothelial Proliferation and Morphogenesis. Oncogene.

[78] Cryan L.M., Rogers M.S. (2011). Targeting the Anthrax Receptors, TEM-8 and CMG-2, for Anti-Angiogenic Therapy. Front. Biosci. Landmark Ed..

[79] Hardenbrook N.J., Liu S., Zhou K., Ghosal K., Zhou Z.H., Krantz B.A. (2020). Atomic Structures of Anthrax Toxin Protective Antigen Channels Bound to Partially Unfolded Lethal and Edema Factors. Nat. Commun..

[80] Balfanz J., Rautenberg P., Ullmann U. (1996). Molecular Mechanisms of Action of Bacterial Exotoxins. Zentralblatt Bakteriol..

[81] Zhang Y. (2015). Why Do We Study Animal Toxins?. Dong Wu Xue Yan Jiu Zool. Res..

[82] Mechaly A., McCluskey A.J., Collier R.J. (2012). Changing the Receptor Specificity of Anthrax Toxin. mBio.

[83] Aktories K. (2022). Treatment of Ovarian Cancer with Modified Anthrax Toxin. Proc. Natl. Acad. Sci. USA.

[84] Jack S., Madhivanan K., Ramadesikan S., Subramanian S., Edwards D.F., Elzey B.D., Dhawan D., McCluskey A., Kischuk E.M., Loftis A.R. (2020). A Novel, Safe, Fast and Efficient Treatment for Her2-positive and Negative Bladder Cancer Utilizing an EGF-anthrax Toxin Chimera. Int. J. Cancer.

[85] Messing E.M. (1990). Clinical Implications of the Expression of Epidermal Growth Factor Receptors in Human Transitional Cell Carcinoma. Cancer Res..

[86] Loftis A.R., Santos M.S., Truex N.L., Biancucci M., Satchell K.J.F., Pentelute B.L. (2020). Anthrax Protective Antigen Retargeted with Single-Chain Variable Fragments Delivers Enzymes to Pancreatic Cancer Cells. ChemBioChem.

[87] Lu Z., Truex N.L., Melo M.B., Cheng Y., Li N., Irvine D.J., Pentelute B.L. (2021). IgG-Engineered Protective Antigen for Cytosolic Delivery of Proteins into Cancer Cells. ACS Cent. Sci..

[88] Bahar M.E., Kim H.J., Kim D.R. (2023). Targeting the RAS/RAF/MAPK Pathway for Cancer Therapy: From Mechanism to Clinical Studies. Signal Transduct. Target. Ther..

[89] Kim J., Harper A., McCormack V., Sung H., Houssami N., Morgan E., Mutebi M., Garvey G., Soerjomataram I., Fidler-Benaoudia M.M. (2025). Global Patterns and Trends in Breast Cancer Incidence and Mortality across 185 Countries. Nat. Med..

[90] Iqbal N., Iqbal N. (2014). Human Epidermal Growth Factor Receptor 2 (HER2) in Cancers: Overexpression and Therapeutic Implications. Mol. Biol. Int..

[91] McCluskey A.J., Olive A.J., Starnbach M.N., Collier R.J. (2013). Targeting HER2-Positive Cancer Cells with Receptor-Redirected Anthrax Protective Antigen. Mol. Oncol..

[92] Panda V.K., Mishra B., Mahapatra S., Swain B., Malhotra D., Saha S., Khanra S., Mishra P., Majhi S., Kumari K. (2025). Molecular Insights on Signaling Cascades in Breast Cancer: A Comprehensive Review. Cancers.

[93] El-Chami D., Al Haddad M., Abi-Habib R., El-Sibai M. (2021). Recombinant Anthrax Lethal Toxin Inhibits Cell Motility and Invasion in Breast Cancer Cells through the Dysregulation of Rho GTPases. Oncol. Lett..

[94] Al-Dimassi S., Salloum G., Saykali B., Khoury O., Liu S., Leppla S.H., Abi-Habib R., El-Sibai M. (2016). Targeting the MAP Kinase Pathway in Astrocytoma Cells Using a Recombinant Anthrax Lethal Toxin as a Way to Inhibit Cell Motility and Invasion. Int. J. Oncol..

[95] Fernando S., Fletcher B.S. (2009). Targeting Tumor Endothelial Marker 8 in the Tumor Vasculature of Colorectal Carcinomas in Mice. Cancer Res..

[96] Sotoudeh M., Shakeri R., Dawsey S.M., Sharififard B., Ahmadbeigi N., Naderi M. (2019). ANTXR1 (TEM8) Overexpression in Gastric Adenocarcinoma Makes the Protein a Potential Target of Immunotherapy. Cancer Immunol. Immunother..

[97] Kareff S.A., Corbett V., Hallenbeck P., Chauhan A. (2023). TEM8 in Oncogenesis: Protein Biology, Pre-Clinical Agents, and Clinical Rationale. Cells.

[98] Sun K.-R., Lv H.-F., Chen B.-B., Nie C.-Y., Zhao J., Chen X.-B. (2021). Latest Therapeutic Target for Gastric Cancer: Anthrax Toxin Receptor 1. World J. Gastrointest. Oncol..

[99] Pietrzyk Ł. (2016). Biomarkers Discovery for Colorectal Cancer: A Review on Tumor Endothelial Markers as Perspective Candidates. Dis. Markers.

[100] Chen K.-H., Liu S., Leysath C.E., Miller-Randolph S., Zhang Y., Fattah R., Bugge T.H., Leppla S.H. (2016). Anthrax Toxin Protective Antigen Variants That Selectively Utilize Either the CMG2 or TEM8 Receptors for Cellular Uptake and Tumor Targeting. J. Biol. Chem..

[101] Moradi F., Ghorbanian N., Hadi N., Khashyar F., Behbahani M.R., Nasrollahian S., Nasoohian N., Jazi N.N., Akbari M. (2025). From Pathogenicity to Therapy: Investigating the Therapeutic Potential of Bacillus Anthracis Anthrax Toxin in Novel Cancer Therapies and Oncological Research. Arch. Microbiol..

[102] Liu S., Wang H., Currie B.M., Molinolo A., Leung H.J., Moayeri M., Basile J.R., Alfano R.W., Gutkind J.S., Frankel A.E. (2008). Matrix Metalloproteinase-Activated Anthrax Lethal Toxin Demonstrates High Potency in Targeting Tumor Vasculature. J. Biol. Chem..

[103] Bachran C., Hasikova R., Leysath C.E., Sastalla I., Zhang Y., Fattah R.J., Liu S., Leppla S.H. (2014). Cytolethal Distending Toxin B as a Cell-Killing Component of Tumor-Targeted Anthrax Toxin Fusion Proteins. Cell Death Dis..

[104] Fischer E.S., Campbell W.A., Liu S., Ghirlando R., Fattah R.J., Bugge T.H., Leppla S.H. (2019). Bismaleimide Cross-linked Anthrax Toxin Forms Functional Octamers with High Specificity in Tumor Targeting. Protein Sci..

[105] Liu S., Aaronson H., Mitola D.J., Leppla S.H., Bugge T.H. (2003). Potent Antitumor Activity of a Urokinase-Activated Engineered Anthrax Toxin. Proc. Natl. Acad. Sci. USA.

[106] Liu S., Ma Q., Fattah R., Bugge T.H., Leppla S.H. (2017). Anti-Tumor Activity of Anthrax Toxin Variants That Form a Functional Translocation Pore by Intermolecular Complementation. Oncotarget.

[107] Telang S., Rasku M.A., Clem A.L., Carter K., Klarer A.C., Badger W.R., Milam R.A., Rai S.N., Pan J., Gragg H. (2011). Phase II Trial of the Regulatory T Cell-Depleting Agent, Denileukin Diftitox, in Patients with Unresectable Stage IV Melanoma. BMC Cancer.

[108] Pemmaraju N., Lane A.A., Sweet K.L., Stein A.S., Vasu S., Blum W., Rizzieri D.A., Wang E.S., Duvic M., Sloan J.M. (2019). Tagraxofusp in Blastic Plasmacytoid Dendritic-Cell Neoplasm. N. Engl. J. Med..

[109] Frankel A.E., Woo J.H., Ahn C., Pemmaraju N., Medeiros B.C., Carraway H.E., Frankfurt O., Forman S.J., Yang X.A., Konopleva M. (2014). Activity of SL-401, a Targeted Therapy Directed to Interleukin-3 Receptor, in Blastic Plasmacytoid Dendritic Cell Neoplasm Patients. Blood.

[110] Weaver M., Laske D.W. (2003). Transferrin Receptor Ligand-Targeted Toxin Conjugate (Tf-CRM107) for Therapy of Malignant Gliomas. J. Neuro-Oncol..

[111] Yang X., Kessler E., Su L.-J., Thorburn A., Frankel A.E., Li Y., La Rosa F.G., Shen J., Li C.-Y., Varella-Garcia M. (2013). Diphtheria Toxin-Epidermal Growth Factor Fusion Protein DAB389EGF for the Treatment of Bladder Cancer. Clin. Cancer Res. Off. J. Am. Assoc. Cancer Res..

[112] Vallera D.A., Chen H., Sicheneder A.R., Panoskaltsis-Mortari A., Taras E.P. (2009). Genetic Alteration of a Bispecific Ligand-Directed Toxin Targeting Human CD19 and CD22 Receptors Resulting in Improved Efficacy against Systemic B Cell Malignancy. Leuk. Res..

[113] Hamlin P.A., Musteata V., Park S.I., Burnett C., Dabovic K., Strack T., Williams E.T., Anand B.S., Higgins J.P., Persky D.O. (2022). Safety and Efficacy of Engineered Toxin Body MT-3724 in Relapsed or Refractory B-Cell Non-Hodgkin’s Lymphomas and Diffuse Large B-Cell Lymphoma. Cancer Res. Commun..

[114] Rezaei Khozani N., Shayesteh Pour M., Yekani M., Hejazi S.H., Saffari M. (2024). Anti-Tumor Effects of Recombinant Clostridium α-Toxin on Breast Cancer: An In Vitro and In Vivo Study. Int. J. Mol. Cell. Med..

[115] Akpınar O., Özşimşek A., Güzel M., Nazıroğlu M. (2020). *Clostridium Botulinum* Neurotoxin A Induces Apoptosis and Mitochondrial Oxidative Stress via Activation of TRPM2 Channel Signaling Pathway in Neuroblastoma and Glioblastoma Tumor Cells. J. Recept. Signal Transduct..

[116] Shim M.K., Na J., Cho I.K., Jang E.H., Park J., Lee S., Kim J.-H. (2021). Targeting of Claudin-4 by Clostridium Perfringens Enterotoxin-Conjugated Polysialic Acid Nanoparticles for Pancreatic Cancer Therapy. J. Control. Release.

[117] Zhang Y., Li Y., Li H., Chen W., Liu W. (2018). Clostridium Difficile Toxin B Recombinant Protein Inhibits Tumor Growth and Induces Apoptosis through Inhibiting Bcl-2 Expression, Triggering Inflammatory Responses and Activating C-erbB-2 and Cox-2 Expression in Breast Cancer Mouse Model. Biomed. Pharmacother..

[118] Dailey K.M., Small J.M., Pullan J.E., Winfree S., Vance K.E., Orr M., Mallik S., Bayles K.W., Hollingsworth M.A., Brooks A.E. (2023). An Intravenous Pancreatic Cancer Therapeutic: Characterization of CRISPR/Cas9n-Modified Clostridium Novyi-Non Toxic. PLoS ONE.

[119] Janku F., Zhang H.H., Pezeshki A., Goel S., Murthy R., Wang-Gillam A., Shepard D.R., Helgason T., Masters T., Hong D.S. (2021). Intratumoral Injection of *Clostridium Novyi*-NT Spores in Patients with Treatment-Refractory Advanced Solid Tumors. Clin. Cancer Res..

[120] Mazor R., Pastan I. (2020). Immunogenicity of Immunotoxins Containing Pseudomonas Exotoxin A: Causes, Consequences, and Mitigation. Front. Immunol..

[121] Simon N., Antignani A., Hewitt S.M., Gadina M., Alewine C., FitzGerald D. (2019). Tofacitinib Enhances Delivery of Antibody-Based Therapeutics to Tumor Cells through Modulation of Inflammatory Cells. JCI Insight.

[122] Abi-Habib R.J., Singh R., Leppla S.H., Greene J.J., Ding Y., Berghuis B., Duesbery N.S., Frankel A.E. (2006). Systemic Anthrax Lethal Toxin Therapy Produces Regressions of Subcutaneous Human Melanoma Tumors in Athymic Nude Mice. Clin. Cancer Res..

[123] Hassan R., Miller A.C., Sharon E., Thomas A., Reynolds J.C., Ling A., Kreitman R.J., Miettinen M.M., Steinberg S.M., Fowler D.H. (2013). Major Cancer Regressions in Mesothelioma After Treatment with an Anti-Mesothelin Immunotoxin and Immune Suppression. Sci. Transl. Med..

[124] Wein A.N., Peters D.E., Valivullah Z., Hoover B.J., Tatineni A., Ma Q., Fattah R., Bugge T.H., Leppla S.H., Liu S. (2015). An Anthrax Toxin Variant with an Improved Activity in Tumor Targeting. Sci. Rep..

[125] Mathew M., Verma R.S. (2009). Humanized Immunotoxins: A New Generation of Immunotoxins for Targeted Cancer Therapy. Cancer Sci..

[126] Liu Z., Jiang W., Nam J., Moon J.J., Kim B.Y.S. (2018). Immunomodulating Nanomedicine for Cancer Therapy. Nano Lett..

[127] Khormi M.A., Al-maaqar S.M., Al Johni A.R., Al-Tayyar N.A., Alhamad J.A., Ghyathuddin A.A., Alblawi Z., Behairy S.M., Alghamdi M.A., Alsubhi W.A. (2025). Oncolytic Bacteria: A Revolutionary Approach to Cancer Therapy. Open Life Sci..

[128] Manning M.L., Mason-Osann E., Onda M., Pastan I. (2015). Bortezomib Reduces Pre-Existing Antibodies to Recombinant Immunotoxins in Mice. J. Immunol..

[129] Antignani A., Sarnovsky R., FitzGerald D.J. (2014). ABT-737 Promotes the Dislocation of ER Luminal Proteins to the Cytosol, Including *Pseudomonas* Exotoxin. Mol. Cancer Ther..

